# Acupuncture for generalized anxiety disorder: a study protocol for a randomized controlled trial

**DOI:** 10.1590/1414-431X2024e13389

**Published:** 2024-05-03

**Authors:** Xiayun Zhou, Guoao Shi, Ruiming Chen, Lingsan Hu, Zhongxian Li, Yifu Zhou, Pan Zhang, Xiang Ji, Min Peng, Kengyu Chen, Luda Yan, Peng Zhou

**Affiliations:** 1 The Seventh Clinical Medical College, Guangzhou University of Traditional Chinese Medicine, Shenzhen, Guangdong Province, China; 2 Shenzhen Bao'an Traditional Chinese Medicine Hospital, Guangzhou University of Chinese Medicine, Shenzhen, Guangdong Province, China

**Keywords:** acupuncture, generalized anxiety disorder, cerebral cortical excitability, transcranial magnetic stimulation, randomized controlled trial, study protocol

## Abstract

During the COVID-19 outbreak, there was a sharp increase in generalized anxiety disorder (GAD). Acupuncture therapy has the advantages of accurate clinical efficacy, safety and reliability, few adverse reactions, and no dependence, and is gradually becoming one of the emerging therapies for treating GAD. We present a study protocol for a randomized clinical trial with the aim of exploring the mechanism of brain plasticity in patients with GAD and evaluate the effectiveness and reliability of acupuncture treatment. Transcranial magnetic stimulation (TMS) will be used to assess cortical excitability in GAD patients and healthy people. Sixty-six GAD patients meeting the inclusion criteria will be randomly divided into two groups: TA group, (treatment with acupuncture and basic western medicine treatment) and SA group (sham acupuncture and basic western medicine treatment). Twenty healthy people will be recruited as the control group (HC). The parameters that will be evaluated are amplitude of motor evoked potentials (MEPs), cortical resting period (CSP), resting motor threshold (RMT), and Hamilton Anxiety Scale (HAMA) score. Secondary results will include blood analysis of γ-aminobutyric acid (GABA), glutamate (Glu), glutamine (Gln), serotonin (5-HT), and brain-derived nerve growth factor (BDNF). Outcomes will be assessed at baseline and after the intervention (week 8). This study protocol is the first clinical trial designed to detect differences in cerebral cortical excitability between healthy subjects and patients with GAD, and the comparison of clinical efficacy and reliability before and after acupuncture intervention is also one of the main contents of the protocol. We hope to find a suitable non-pharmacological alternative treatment for patients with GAD.

## Introduction

The long-term aspect and repeated outbreaks of COVID-19 have had a serious impact on China's economy and life. In general, emotional problems are the most prominent effects of experiences and feelings in the face of public health emergencies, with common manifestations of psychological symptoms such as anxiety and panic (1,2). A clear correlation has been observed between the prevalence of anxiety disorders and the new coronavirus infection (3). Isolation measures and other factors contributed to the problem (4). In 2020, approximately 374 million individuals worldwide were afflicted with anxiety disorders, out of which around 76.2 million cases were attributed to the epidemic itself. Furthermore, there has been a notable surge of 25.6% in the number of patients suffering from anxiety disorders since the advent of this pandemic in 2020 (3,5). Generalized anxiety disorder (GAD), as the most common type of anxiety disorder (6), is characterized by tension, worry, fear, and autonomic nervous dysfunction (7). It is a chronic disabling disease with a low rate of complete remission and difficult treatment (8). Persistent mental and physical symptoms not only seriously interfere with the daily life, study, work, and social interaction of GAD patients, but also seriously impair their physical and mental health (9).

At present, drug therapy is the preferred treatment for GAD (10). Benzodiazepine anti-anxiety drugs, non-benzodiazepine anti-anxiety drugs, receptor blockers, selective 5-HT reuptake inhibitors, etc. are common Western medicine drugs, but the long-term use of such drugs can lead to dependence, addiction, and increased incidence of drug adverse reactions and drug resistance, reducing the therapeutic effect (11). Therefore, there is an urgent need for a treatment that is effective, safe, and easy to promote. As a mature and suitable technique of traditional Chinese medicine (TCM), acupuncture therapy has gradually become one of the emerging therapies for GAD.

At the present stage, clinical and, especially, mechanistic studies of acupuncture intervention are quite limited, and only a few scholars have made preliminary exploration. Here, we present the protocol of a clinical trial that aims to confirm the effectiveness and reliability of acupuncture therapy in the treatment of GAD patients and explore the relevant mechanisms to facilitate the further application of acupuncture therapy.

## Material and Methods

### Study design

**Figure 1:**
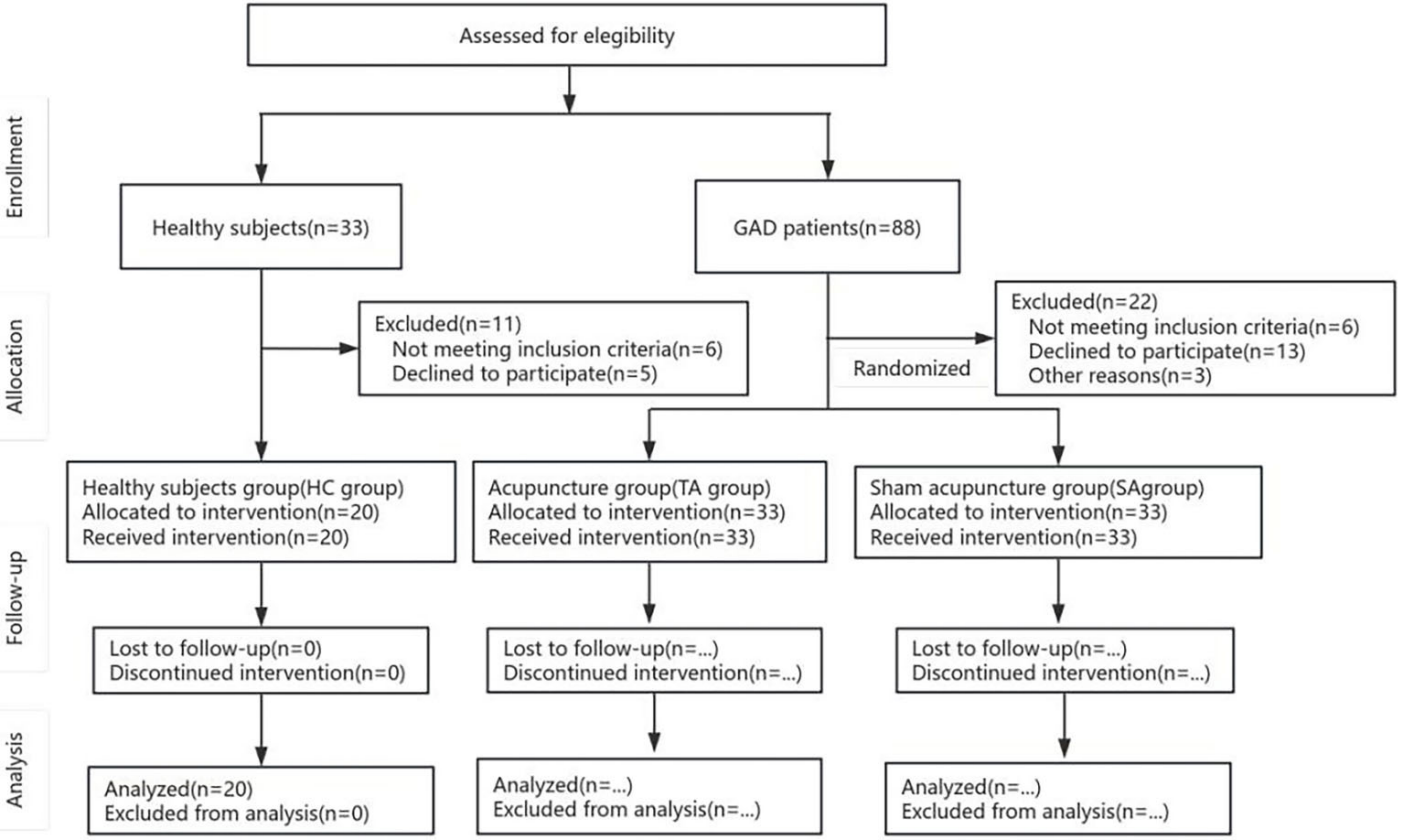
Study flow chart, in accordance with the CONSORT guidelines.

The study will be a randomized, parallel-controlled clinical trial. The main purpose of the study will be to explore the changes of cortical excitability in patients with GAD and evaluate the effectiveness and reliability of acupuncture treatment to GAD. The study will enroll 66 GAD patients who meet the inclusion criteria, and they will be randomly divided at a 1:1 ratio into the TA group (treatment with acupuncture) and the SA group (sham acupuncture). In addition, 20 healthy people will be recruited as the control group (HC group). For ethical reasons, patients from both the TA and SA groups will receive basic western medicine treatment with paroxetine hydrochloride. We will explore whether there are cortical excitability changes between GAD patients and the healthy people by comparing the relevant indicators of transcranial magnetic stimulation (TMS) and evaluate the efficacy of acupuncture by comparing the relevant indicators before and after treatment between the TA group and the SA group. The flowchart of this protocol is shown in Figure 1.

### Patients with GAD

#### Inclusion criteria

Diagnosis of GAD according to the CCMD-3 Chinese Classification and Diagnostic Standards for Mental Disorders formulated by the psychiatric branch of the Chinese Medical Association and the Fifth Diagnostic and Statistical Manual of Mental Disorders in the US and having been diagnosed during COVID-19 and having had a quarantine event; Hamilton Anxiety Scale (HAMA, 14 items) score ≥14 points and <29 points; Age from 18 to 65 years old (unrestricted sex, right-handed); No treatments within the past three weeks, including drug, psychological, or physical treatment such as transcranial magnetic therapy or other acupuncture treatments; Stable condition, no self-harming or suicidal behavior; Voluntary participation and signature of the informed consent prior to inclusion.

#### Exclusion criteria

Patients who do not meet the inclusion criteria; Patients with a previous history of brain trauma, epilepsy, metal implants, cardiovascular and cerebrovascular diseases, liver, kidney, and other serious somatic and organic diseases; Patients with schizophrenia, bipolar disorder, or other mental disorders or physical diseases that can show symptoms of anxiety; Anxiety due to past and present self-injury, suicidal plan or behavior, psychotic symptoms, or alcohol and drug addiction; Poor compliance of acupuncture treatment or fear of acupuncturists; Left-handed; Women with pregnancy and lactation.

#### Termination criteria

Serious adverse reactions or events and patient cannot continue with the treatment; During the course of the study, the subject has serious combined disease or serious disease of other system and cannot continue with the treatment; The subject has poor compliance and does not cooperate with the assigned treatment after repeated explanation by the doctor; The participant requests to withdraw from the study; The participant takes other therapeutic drugs independently during the treatment period, which may interfere with research results; Patients lost to follow-up for various reasons.

### Healthy people

#### Inclusion criteria

Aged between 18 and 65 years; Right-handed, any gender; Physical health and no physical discomfort in the past month; No central nervous system stimulation such as repeated transcranial magnetic stimulation (rTMS), transcranial direct current stimulation (TDCS), or peripheral stimulation such as acupuncture and percutaneous electrical stimulation; No history of illicit drug use and excessive alcohol consumption; Signing of the informed consent form and volunteer participation in the investigation.

#### Exclusion criteria

People with speech disorders; Hypertension, diabetes mellitus, and major diseases of the heart, liver, kidney, and other organs; History of dementia, mental illness, palsy, and other neurological diseases; History of craniocerebral trauma and surgery; Metal artifacts in the body (including dentures, pacemakers, neurostimulators, medical pumps, etc.); TMS fear and other reasons for avoiding TMS stimulation; Women with pregnancy and lactation.

#### Termination criteria

Motor cortex area not detected by TMS; Fail to receive the treatment within the prescribed time due to personal reasons; Poor compliance and no cooperation with the researcher; Personal withdrawal from the study.

### Study setting

**Figure 2:**
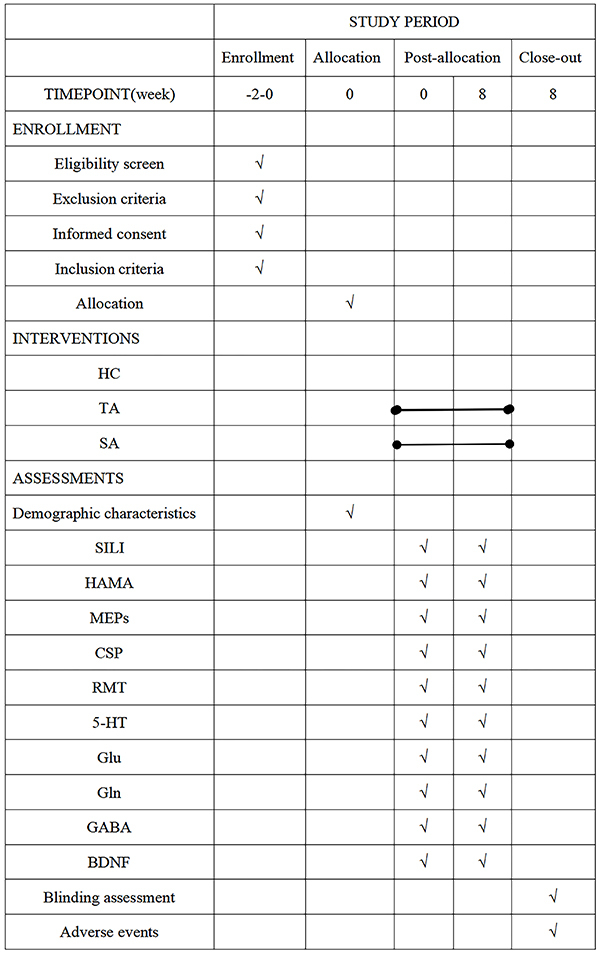
Enrollment, interventions, and assessments. The line segments in TA and SA in the graph indicate the duration of treatment. HC: healthy control group; TA: treatment with acupuncture group; SA: sham acupuncture group; SILI: safety index laboratory inspection; HAMA: Hamilton Anxiety Scale; MEPs: amplitude of motor evoked potentials; CSP: cortical resting period; RMT: resting motor threshold; 5-HT: 5-hydroxytryptamine; Glu: glutamic acid; Gln: glutamine; GABA: γ-aminobutyric acid; BDNF: brain-derived neurotrophic factor.

The study team will recruit subjects from the general public through the Publicity Department of Bao'an Hospital of Traditional Chinese Medicine affiliated to Guangzhou University of Traditional Chinese Medicine. The treatment will be conducted in the Acupuncture Branch of that hospital. In total, 86 people will be recruited for this study, 33 in each treatment group and 20 in the healthy control group. Patients enrolled in the study will be required to sign an informed consent form and participate in the study on a voluntary basis. The timeline for patient enrollment, intervention, and assessment is shown in Figure 2.

### Recruitment

We will recruit patients from the acupuncture department, acupuncture ward, psychological clinic, insomnia outpatient department and subordinate social health, general clinic of Bao'an Hospital of Traditional Chinese Medicine in Shenzhen, with COVID-19 quarantine experience, and who are diagnosed with GAD. All the participants will participate in a pre-enrollment information session about the treatment and assessments to ensure subject data retention and completion of follow-up. A WeChat group will be established for all participants to communicate with doctors and report adverse events at any time.

### Randomization

Baseline data from the participants will be collected and evaluated before enrollment. Healthy people will be numbered in the order of enrollment, while the GAD patients will be randomly assigned to the TA group and SA group at a 1:1 ratio. To ensure a random distribution, randomization will be carried out by the Institute of Acupuncture, which will not participate in this study. The assigned random numbers and grouping results will be kept in opaque envelopes, which will be kept by the staff not enrolled in this study and later opened by a research assistant who will assign participants according to the order of patient enrollment.

### Blinding

In this study, the acupuncturists cannot be blinded due to the particularity of the acupuncture treatment, but the participants and the statisticians will be blinded. Treatment, testing, and follow-up will be performed independently without interaction between the groups. At the end of 8 weeks, participants in the acupuncture and sham acupuncture groups will be asked the following questions to test for blinding: “Are you confident with this treatment?” “Do you accept this type of acupuncture?” Participants will choose either “Yes” or “No” to answer the questions. These data will be analyzed at the end of the study. If the results are not significant, then it can be concluded that blinding was reliable. To ensure the consistency of treatment, the treatment and testing will be completed by the same physician. The collectors of the data, the staff, and the statisticians will not have access to the assigned tasks.

### Intervention

**Figure 3:**
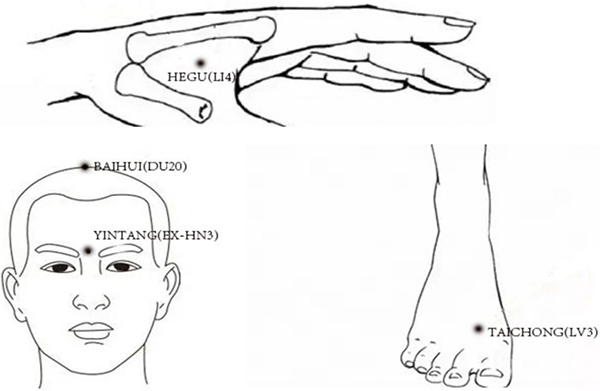
The six acupoints used in the study: BAIHUI (DU20), YINTANG (EX-HN3), HEGU (LI4) (both sides), and TAICHONG (LV3) (both sides). Two are applied on both sides.

The protocol was designed based on previous clinical studies and basic theories of TCM (12). All procedures complied with the Acupuncture Clinical Trial Interventions Reporting Standards (STRICTA) (13). According to the WHO Standard and Acupoint Guidelines (14), acupoints applied in the two groups will be the same, which are listed in Table 1 and Figure 3. All the acupuncturists in the study must have over 3 years of relevant work experience. Before implementation, all the researchers will be uniformly trained for this study. The HC group will not receive any treatment, while two groups of GAD patients will receive 8 weeks of acupuncture treatment for a total of 24 sessions. Patients will be asked to not use drugs or other treatments that affect the study or have any other acupuncture treatment so as not to interfere with the study evaluation of the effectiveness in the study.

**Table 1:** Acupoints used in both the treatment with acupuncture (TA) group and the sham acupuncture (SA) group.

Name	Location
YINTANG (EX-HN3)	At the forehead, mid-point between the two eyebrows.
BAIHUI (DU20)	On the median line of the head, 5 in. superior to the anterior hairline, at about the middle of the connecting line between both auricular tips.
HEGU (LI4)	On the back of the hand, between the first and second metacarpal bones, at the midpoint of the radial side of the second metacarpal bone.
TAICHONG (LV3)	On the dorsum of the foot, between the first and second metatarsal bones, in the depression in front of the metatarsal joint, or touching the artery where the pulse can be felt.

### TA group

Before undergoing acupuncture treatment, the patient will be asked to lie in the flat supine position in a quiet environment. The acupuncturist will sterilize the skin near the selected acupoints with 0.5% tincture of iodine or 75% alcohol, then they will choose a one-time use stainless steel needle (25×25 mm, Huatuo Suzhou, China) and insert it into the skin quickly. Needles will be inserted in the YINTANG (EX-HN3), BAIHUI (DU20), HEGU (LI4), and TAICHONG (LV3) points and twirled gently to “Deqi”, which is a therapeutic response to acupuncture that refers to the sense of qi: the patient feels a heavy and astringent feeling under the needle, with numbness, swelling, or heavy feeling. After that, the needle will be retained for 30 min. The specific depth of acupuncture depends on individual factors. When the needle is removed, the acupuncturist will press the needle hole with a cotton swab for about 1 min to prevent bleeding. Acupuncture treatment will be performed once a day, 3 times a week for 8 weeks.

### SA group

The environment of the treatment, acupuncture location, and treatment frequency of the SA group will be consistent with those of the TA group. The acupuncturist will choose a blunt needle (20×25 mm, Huatuo Suzhou) for the SA group and attach a translucent tube to the same acupoints as in the TA group. Then, the acupuncturist will insert a blunt needle into the tube and hold the needle upright. The patient will feel a mild stabbing sensation, but the needle will not penetrate their skin.

### Basic western medicine

Paroxetine hydrochloride tablets (Leyou, 20 mg/day, 30 tablets per box) will be prescribed to patients of the two treatment groups at the beginning of acupuncture treatment and they will be required to take the medication under the guidance of our psychologist.

### HA group

Healthy controls will not undergo any intervention.

### Outcome

TMS and biochemical measures will be used as measures of outcome. The main observations include the amplitude of motor-evoked potentials (MEPs), cortical resting period (CSP), resting motor threshold (RMT), and Hamilton Anxiety Scale scores (HAMA), while serotonin (5-HT), glutamate (Glu), glutamine (Gln) in peripheral serum, and γ-aminobutyric acid (GABA) and brain-derived nerve growth factor (BDNF) will be used as secondary outcomes. Biospecimens will be kept at -80°C after collection. All biological specimens will be analyzed in this assay and used in future ancillary studies.

### Primary outcomes

The primary outcomes of this study will be the HAMA and the single-pulse TMS measures. The HAMA scale comprises 14 items, and assessment before and after treatment is conducted by specialized psychiatric physicians. The HAMA total score ranges from 0 to 56, with anxiety levels categorized as follows: possible anxiety (8≤ total score ≤14), definite anxiety (14< total score ≤21), moderate anxiety (21< total score ≤29), and severe anxiety (>29). Single-pulse TMS is a common method for assessing changes in motor cortex excitability. In this study, the key TMS parameters will include the amplitude of MEPs, CSP, and RMT. The Magstim Rapid2 transcranial magnetic stimulation device from Magstim (UK) and the Nicolet Viking Quest physiological recorder from Natus Neurology Incorporated (USA) will be employed for measurements. Single-pulse TMS will be applied with a maximum output intensity of 2.2 T, corresponding to the first dorsal interosseous muscle. Changes in data will be assessed before and after treatment for all participants including the RMT of the motor cortex, the MEP-A, and the CSP. Efforts will be made to ensure that assessments before and after treatment for all patients are conducted as close as possible to the same time period.

### Secondary outcomes

GABA and Glu, as the main inhibitory and excitatory neurotransmitters in the brain, play a crucial role in the regulation and control of bilateral cerebral cortex excitability, and the imbalance between them is considered to be the basis of mental health diseases (15). Gln is converted from Glu, 5-HT is the main substance to control emotions, and BDNF has a high impact on brain plasticity. The quantitative detection of the above five peripheral blood indicators before and after treatment will be used to evaluate the efficacy of the treatment. All blood samples will be collected from participants in a calm state on an empty stomach before 9 a.m.

### Sample size

The sample size for this study was estimated using the statistical data analysis software PASS15 (NCSS Statistical software, https://www.ncss.com/support/). The parameters Z1-α and Z1-β were consulted from tables, where πT represents the efficacy rate in the observation group, πC represents the efficacy rate in the control group, and K is the ratio of the number of cases in the treatment group to the control group, set at 1. Due to the current lack of mechanistic studies on neuroplasticity in patients with GAD and the absence of high-quality clinical research, we established, based on literature review (16,17) and preliminary trials, a control group efficacy rate of 70% and a treatment group efficacy rate of 96%. A maximum acceptable difference of 15% in efficacy rates between the observation and control groups was considered.

Through computations using the PASS15 software with parameters α=0.05 and β=0.2, it was determined that a minimum of 29 cases per group was required. Since the control group of healthy individuals does not require treatment, dropout issues were temporarily disregarded. A total of 58 GAD subjects were required. Assuming a ratio of 1:3 between the healthy and GAD patient groups, approximately 20 individuals in the healthy group were deemed necessary. Considering a potential dropout rate of 10% in both groups receiving acupuncture treatment for GAD, each group should include 33 participants, resulting in a total of 66 participants.

### Safety assessment

To reduce the risk of adverse events, acupuncturists participating in the study will be required to have not less than 3 years of clinical work experience. Before the start of the study, all the acupuncturists will be uniformly trained to standardize the acupuncture operations to increase the safety and synergistic comparability of the acupuncture. A psychologist will assess the patient's condition to ensure that the patients with severe psychological disorders can be promptly treated during the study. The acupuncturist also will evaluate the condition during treatment to reduce the occurrence of adverse events. Furthermore, this study will be conducted in Shenzhen Bao'an District Hospital of Traditional Chinese Medicine, which has a high medical level and the ability to handle crisis events and can guarantee the progress of this study.

Before recruitment and randomization, all participants will undergo routine blood and urine tests, as well as liver and kidney function assessments, to identify and exclude individuals with severe heart, liver, or kidney diseases. Possible side effects during the intervention and potential adverse reactions to acupuncture, including minor local bleeding at the needling site, redness, palpitations, dizziness, headaches, nausea, vomiting, and pain, will be carefully monitored. All adverse events occurring in the study will be recorded using the Universal Reporting Form (URF). Researchers will promptly manage and record the adverse events. If a serious adverse event occurs, it will be reported to the Ethics Committee of Shenzhen Bao'an District Hospital of Traditional Chinese Medicine immediately. Participants who are unable to continue their participation in the study due to adverse events will be excluded.

### Data collection and management

This study will collect basic information about eligible participants before the first treatment, including name, age, gender, occupation, and course of disease. During treatment and follow-up, medication and acupuncture reactions will be recorded according to the participants' response. These data will be collected at weeks 0 and 8. Data collection will be performed by three staff members who are not involved in acupuncture treatment, index evaluation, statistical analysis, or grouping. Data collection and entry work will be carried out independently by two staff members and will be supervised and inspected by a third staff member. Finally, they will complete the above work together. Principal researchers, acupuncturists, TMS operators, and laboratory staff will not be involved in data collection.

All data will be kept in paper documents and stamped with identification codes, the electronic data will be stored on the clinical trial management public platform ResMan Research Manager, and photographs will be named using identification codes to ensure authenticity and integrity. All data will be kept for at least 5 years. A data monitoring committee composed of experts with good clinical research experience from the acupuncture Department of Shenzhen Bao'an District Hospital of Traditional Chinese Medicine will regularly monitor research progress, data, participant management, distribution, and more. The Committee will be independent of the funding sponsor to avoid conflict of interest. The researchers responsible for data collection and entry will have access to the interim results and report to the principal investigator if necessary. The principal investigators will make a final decision to terminate the trial if so is decided after discussion.

### Quality control

Before the study, all participating researchers will undergo uniform training. The investigators should fully understand the purpose and nature of this study. All acupuncturists must have a licensed medical certificate and 3 years of relevant clinical work experience. Prior to the study, all acupuncturists will receive uniform training to clarify the acupuncture criteria in this study. The principal investigator will examine the URF weekly during the study. This study data will be collated and analyzed by specific staff members, with no work overlap among them. Adverse events during the study will be documented in detail. The personal data of the participants will be kept by the researcher, and no one else will have access the relevant information.

### Statistical analysis

This study will use the principles of protocol-by-agreement (PP) and intention-to-treat (ITT) for analysis. The PP principle will analyze the data of all participants who complete the study protocol, and individuals who do not complete the protocol will not be included. ITT analysis will include all subjects who have attended at least one treatment, including those who have dropped out. For participants who exit, the missing data will be supplemented with the latest data.

The data in this study will be analyzed by professional statisticians using SPSS 26.0 software (IBM SPSS Statistics, IBM Corp, USA). Firstly, we will describe baseline data, such as age, course of disease, and sex, and compare baseline data between the three groups. If continuous variables conform to a normal distribution, they will be reported as the mean±SD, otherwise they will be reported as the median (P25,P75). Categorical variables will be reported as number and percentage.

Analysis of variance will be used for continuous variables conforming to normal distribution, and rank sum test will be used for analysis of data not conforming to normal distribution. In addition, we will analyze the categorical variables using chi-squared test or Fisher's exact test. The confidence interval (CI) will be estimated at 95%, and the significance level was set at 0.05. The relationship between side effects and acupuncture treatment will be analyzed. We will also calculate the drop-out rate and analyze the reasons for drop-out.

### Ethics and dissemination

The study was designed according to the Declaration of Helsinki, approved by the Institutional Ethics Committee of Bao'an District Hospital of Traditional Chinese Medicine, Shenzhen City (approval No. KY-2022-035-01), and registered in Chinese Clinical Trial Registry (registration number: ChiCTR2200066311). As this study protocol is part of the registration program, the observational indicators will not be completely consistent. However, the study population, intervention measures, control group, observational indicators, and design have not been specially modified. In addition, if there are any changes in the clinical study protocol and informed consent, the investigators will be required to promptly report the changes to the Ethics Committee and Registry.

Researchers should fully inform patients of the treatments and tests that need to be completed before subjects are enrolled in the study, as well as the possible risks of the study and their right to withdraw from the study. Patients should also be informed of the possible adverse reactions to the collection of therapeutic or biological specimens. To protect the subjects' rights and interests, all treatments and tests in this study will be provided free of charge, and all participants will be provided with free medical advice and guidance. Participants will also receive part of the cost of travel to and from the hospital, all of which will improve participant compliance.

## Results

### Demographic characteristics

Baseline characteristics of the TA and SA groups included gender, age, height, weight, marital status, degree of education, occupation and whether they had recurrent GAD. There were no statistically significant differences between the two groups at baseline, as shown in Table 2. At the same time, we recruited 20 healthy people with no statistically significant differences in age (t=1.397, P=0.168) and sex (X^2^=0.077, P=0.781) compared to the 66 GAD patients as controls.

**Table 2:** Baseline characteristics of the treatment with acupuncture (TA) and sham acupuncture (SA) groups.

Variable	TA group (n=33)	SA group (n=33)	P-values
Gender*			0.459
Male/female	17 (51.52%)/16 (48.48%)	14 (42.42%)/19 (57.58%)	
Age/year***	37.00 (27.00, 44.00)	31.00 (26.00, 39.00)	0.156
Height/cm**	166.67±6.08	164.91±6.95	0.310
Weight/kg***	60.00 (52.00, 65.00)	56.00 (51.50, 61.50)	0.218
Course of the disease/month***	30.00 (18.00, 63.00)	24.00 (12.00, 45.00)	0.240
Marital status*			0.955
Married	18 (54.55%)	10 (30.30%%)	
Unmarried	14 (42.42%)	21 (63.64%)	
Divorced	1 (3.03%)	2 (6.06%)	
Degree of education*			0.955
High school and below	5 (15.15%)	4 (12.12%)	
Junior college	7 (21.21%)	7 (21.21%)	
Undergraduate course	15 (45.45%)	17 (51.52%)	
Graduate student or above	6 (18.18%)	5 (15.15%)	
Occupation*			0.789
Office worker	9 (27.27%)	12 (36.36%)	
Professionals	5 (15.15%)	2 (6.06%)	
Business service personnel	8 (24.24%)	8 (24.24%)	
Liberal professions	6 (18.18%)	8 (24.24%)	
Student	2 (6.06%)	1 (3.03%)	
Other	3 (9.09%)	2 (6.06%)	
Whether is recurrent GAD*			0.084
No/Yes	12 (36.36%)/21 (63.64%)	19 (57.58%)/14 (42.42%)	

Data are reported as number (%); chi-squared test. **Data are reported as means±SD; independent Student's *t*-test. ***Data are reported as median (P25, P75); Mann-Whitney U test. GAD: generalized anxiety disorder.

### Trial status

The trial is currently in the phase of treatment implementation. The agreement was registered on December 01, 2022, with code ChiCTR2200066311. From December 2, 2022 to December 25, 2023, we completed the recruitment of all subjects in this study protocol, of which 20 healthy subjects are undergoing TMS testing and blood tests, and all subjects with GAD are being treated and tested in the study protocol. If we should amend the protocol, we will communicate with the investigators, ethics committee, and trial registries.

## Discussion

In the context of the COVID-19 pandemic, there has been a significant increase in global patients suffering from GAD. Acupuncture, as a non-pharmacological intervention rooted in TCM, has gradually emerged as a suitable, safe, and effective alternative for treating anxiety disorders (18). However, there is currently limited clinical research on acupuncture for treatment of GAD, and the mechanisms underlying its anxiolytic effects remain unclear.

Multiple imaging studies (19-21) have confirmed that the activity of the prefrontal lobe of patients with GAD is decreased compared with that of healthy people, which may be due to the top-down control defect of the prefrontal lobe over the amygdala leading to emotional regulation disorder, and the severity of anxiety is negatively correlated with the activation degree of the prefrontal lobe. This suggests that there may be abnormal changes in the cortical excitability of GAD patients or a pathogenesis hypothesis of abnormal brain plasticity.

The MEP latency and central motor conduction time are considered indicators of the integrity of the cortical-spinal pathway, while MEP amplitude is utilized to measure the excitability state of neurons connecting the motor cortex and muscles (22). According to the recommendations of the International Federation of Clinical Neurophysiology (23), RMT is regarded as a global parameter of brain excitability, as it represents a composite measurement of membrane excitability. CSP can be employed for functional assessment of inhibitory circuits within the cortex (24). These indices serve as crucial markers for assessing the excitability of the cerebral cortex and are important indicators of brain plasticity.

The dorsolateral prefrontal cortex (DLPFC) is a crucial cortical structure responsible for regulating emotions and other higher cognitive functions (25). Multiple studies have confirmed abnormal DLPFC activity in individuals with GAD during psychological tasks (26-29), suggesting a potential intimate relationship between the prefrontal cortex and emotional regulation. In this study, leveraging the detection capabilities of TMS, the left DLPFC associated with negative emotions was chosen as the targeted region for assessment. We aimed to measure changes in cortical excitability, explore the relationship between GAD and brain plasticity, and, in conjunction with relevant biochemical markers, delve into the clinical efficacy mechanisms. This research strived to further clarify the effectiveness and reliability of acupuncture treatment.

We hope that the data analysis obtained after the completion of this study will provide a robust data basis for subsequent research on the mechanism of acupuncture in the treatment of GAD and the wide application of large-sample treatment.

### Comprehensive selection of outcome measures

TMS is a magnetic stimulation technology that acts on the central nervous system, which can change the membrane potential of cortical nerve cells through a time-varying pulsed magnetic field, thus generating induced current, affecting the electrical activity of nerves and brain metabolism, and then causing a series of physiological and biochemical reactions. Monopulse TMS is commonly used to evaluate changes in motor cortex excitability.

Single pulse stimulation of the motor cortex, spinal cord nerve roots, or peripheral nerves of the brain can not only reflect the function of the central motor conduction pathway, but also evaluate the degree of motor neuron damage by recording the amplitude and latency changes of the action evoked potential at the target muscle. Therefore, single pulse stimulation is widely used in the field of electrophysiological research, such as clinical detection of corticospinal cord excitability changes. By recording the amplitude and latency of MEP generated by stimulation, important parameters of the functional state of corticospinal tract can be evaluated, and the intensity of stimulation can be judged by the MEP generated, so as to reflect the changes in the excitability of motor cortex (30), which can provide reliable information for the physiology and pathology of motor conduction function (31). The main indicators selected for TMS in this study included MEPs, CSP, and RMT, which can be used to evaluate cortical changes in GAD patients.

BDNF is involved in activity-dependent neuroplasticity, including regeneration, repair, and protection after injury. GABA and Glu, as the main inhibitory and excitatory neurotransmitters in the brain, are interrelated and mutually restricted, and they play a crucial role in regulating and controlling the excitability of bilateral cerebral cortex (15). The abnormal functions of both are related to the pathogenesis of GAD (32). Gln is the main intermediate of GLU recycling, and the two are always in equilibrium. 5-HT, which is found in higher concentrations in brain tissue, is an important substance that regulates neural activity. In this study, we will observe whether acupuncture plays an anxiolytic role by regulating the level of the above indicators.

### Selection of medication and placebo acupuncture method

In previous acupuncture studies, other sham acupuncture methods were used, such as shallow skin acupuncture, non-point acupuncture, random point acupuncture, etc. (33). Considering the effectiveness of acupuncture for anxiety disorders, blunt needle acupuncture was adopted, because this study mainly explored the evaluation of the efficacy of acupuncture for soothing the liver and regulating the mind. In this way, the effect of epidermal acupuncture could be avoided, and the design would be more rigorous.

In terms of drug selection, this study will use paroxetine hydrochloride tablets, which is the most effective and selective serotonin reuptake inhibitor (SSRIs) (34). This is an emerging and commonly used anxiolytic drug in clinical practice, which can inhibit the reabsorption of 5-HT and increase the concentration of 5-HT, thus achieving anti-anxiety effects. Due to ethical considerations and the rigor of the study design, GAD patients will be given an oral dose of 20 mg per day under the guidance of a psychologist.

### Limitations of the study

In the clinical implementation process, this study was limited by manpower and funds, and the time points of TMS detection before and after treatment were not completely consistent for only a few subjects. This study was designed to explore and evaluate the efficacy and mechanism of acupuncture in the treatment of GAD. Because the research is still in the exploration stage, we have not paid detailed attention to the influence of the degree of anxiety on brain plasticity, and we hope to further improve and deepen the research in the future.
